# Analyzing Brain Connectivity in the Mutual Regulation of Emotion–Movement Using Bidirectional Granger Causality

**DOI:** 10.3389/fnins.2020.00369

**Published:** 2020-05-06

**Authors:** Ting Li, Guoqi Li, Tao Xue, Jinhua Zhang

**Affiliations:** ^1^Shaanxi Key Laboratory of Clothing Intelligence, School of Computer Science, Xi’an Polytechnic University, Xi’an, China; ^2^State and Local Joint Engineering Research Center for Advanced Networking & Intelligent Information Services, School of Computer Science, Xi’an Polytechnic University, Xi’an, China; ^3^Center for Brain Inspired Computing Research (CBICR), Tsinghua University, Beijing, China; ^4^State Key Laboratory for Manufacturing Systems Engineering, Key Laboratory of Education Ministry for Modern Design and Rotor-Bearing System, Xi’an Jiaotong University, Xi’an, China

**Keywords:** emotion, movement, bi-LSTM network, Granger causality (GC), EEG, brain connectivity analysis

## Abstract

Body language and movement are important media of emotional expression. There is an interactive physiological relationship between emotion and movement. Thus, we hypothesize that the emotional cortex interacts with the motor cortex during the mutual regulation of emotion and movement. And this interaction can be revealed by brain connectivity analysis based on electroencephalogram (EEG) signal processing. We proposed a brain connectivity analysis method: bidirectional long short-term memory Granger causality (bi-LSTM-GC). The theoretical basis of the proposed method was Granger causality estimation using a bidirectional LSTM recurrent neural network (RNN) for solving nonlinear parameters. Then, we compared the accuracy of the bi-LSTM-GC with other unidirectional connectivity methods. The results demonstrated that the information interaction existed among multiple brain regions (EEG 10-20 system) in the paradigm of emotion–movement regulation. The detected directional dependencies in EEG signals were mainly distributed from the frontal to the central region and from the prefrontal to the central–parietal.

## Introduction

The preface to the Book of Songs contains the following quote: “Feelings are in the middle and words are in the form. The lack of words makes us lament it. Sigh at the inadequacy of the song forever. The inadequacy of the eternal song, not knowing the dance of the hand, is also the dance of the foot.” Emotion and movement are independent cognitive systems, but they also feature an interrelated bidirectional regulation. Such natural parallelism among multiple cognitive functions of the human brain is known as *synesthesia* (Gazzaniga et al., 2014). We used electroencephalogram (EEG) analysis to find brain connectivity evidence for movement–emotion regulation.

Recently, many psychological, neurological, and robotic studies have focused on movement–emotion regulation from different perspectives. Body language is a powerful form of non-verbal communication that can provide important clues for understanding the intentions, emotions, and motivations of others (Tipper et al., 2015). The mirror neuron theory (Cattaneo and Rizzolatti, 2009) explains the fundamental neurophysiological mechanisms of movement observation and related emotion recognition. Several studies have shown that damage to the inferior frontal gyrus or dorsal lateral prefrontal cortex regions can cause deficits in both emotion recognition and movement control, with EEG showing less mu suppression in the sensorimotor cortex (Perry et al., 2017). In another study, afferent muscle firing was modified by a sad emotional context, with increases in the muscle spindle dynamic response (Ackerley et al., 2017).

Emotion–motion regulation is a complex cognitive process. Studies have shown that, when body language, movement control, and emotional regulation are integrated, multiple brain function modules participate in cooperative information processing (Weis and Herbert, 2017; Melzer et al., 2019). To study this problem, researchers must investigate information exchange throughout multiple brain regions. By processing brain signals at different levels and extracting the characteristics of these signals, researchers have explored the neural mechanisms of cognitive processes in order to identify methods to decode these processes (Babiloni et al., 2017; Yu et al., 2017; Filho et al., 2018).

Brain connectivity analysis aims to characterize information propagation relationships among multiple neural signal channels to explain the underlying structural and functional relationships that exist among various brain regions (Bernhardt et al., 2013). Such relationships include anatomical connectivity, functional connectivity, and effective connectivity (causal interactions). Effective connectivity provides information about the causal (directional) interactions between one neural element or brain region and another. Some techniques for ascertaining effective connectivity require a model that follows certain specifications and structural parameters (Honey et al., 2007).

Granger causality (GC) is largely a model-based method for making time series causality measurements. Such methods involve a large computational burden and many *a priori* assumptions. In this regard, feed-forward neural networks are the most common statistical tools used to carry out non-parametric regression (Montalto et al., 2015), and these tools could be used to solve the problems of the GC approach. Montalto et al. (2014) proposed that neural networks could be used to solve model parameter regression in GC analysis, claiming that the method could realize the Granger paradigm in a non-parametric pattern. Wang et al. (2018b) proposed a GC measurement method based on the long short-term memory (LSTM) recurrent neural network (RNN). Their method involved an estimator that solved the problem of variable-length time lags and long transmission delays.

The functional distribution of the emotional cortex differs markedly from that of the motor cortex, with some spatial coupling. The main brain regions involved in emotion processing are the dorsal prefrontal cortex, the ventral medial prefrontal cortex, the orbitofrontal cortex, the amygdala, the hippocampus, the anterior cingulate cortex, and the insular cortex (Gazzaniga et al., 2014). The prefrontal cortex refers to the whole frontal cortex, except the primary and secondary motor cortex, and plays a key role in the generation and regulation of emotions (Gazzaniga et al., 2014). The EEG signal shows many complex and nonlinear characteristics. Through the scalp, EEG measures the sum of a large number of synaptic postsynaptic potentials synchronously. It is an overall reflection of the electrophysiological activities of neurons in the cerebral cortex or scalp surface. Neuron discharge and transmission are complex processes. Time-dependent complexity is an important manifestation of the nonlinear characteristics of EEG. Bidirectional RNN could be used to solve the problem of bidirectional time dependency. We propose that current EEG signals are related to both previous and future signals. It follows that a bidirectional dependency evaluation algorithm is needed to effectively evaluate the complex, nonlinear temporal dependency of EEG signals on the synchronous discharge of a large number of neurons. To analyze brain connectivity related to emotion–motion regulation, we combined the bidirectional RNN (bi-LSTM) model and GC to create a bi-LSTM-GC time series causality analysis algorithm.

In the present study, the main objective was to discover brain connectivity evidence for emotion–movement regulation using EEG analysis. The bi-LSTM-GC algorithm was proposed to deal with the problem of complex temporal dependency in EEG signals. The experimental data of emotion–movement regulation showed directional dependencies, mainly from frontal to central and from prefrontal to central–parietal.

## Materials and Methods

### Dataset

#### Stimulation by Music (DEAP Dataset)

The DEAP is a publicly available dataset for emotion analysis (Koelstra et al., 2012). It was compounded by recording the EEG signals and peripheral physiological signals of 32 participants. To this end, 32 active AgCl electrodes were used. The sampling rate was 512 Hz, with the data then being down-sampled to 128 Hz. The valence–arousal model was used to calibrate the parameters of the emotional states. Participants watched 40, 1 min long excerpts of music videos. They then selected the numbers 1–9 to indicate their emotion states and rated each video in terms of the levels of arousal and valence. The valence–arousal space was subdivided into nine quadrants, each with a tag (V–A tag), namely, low arousal/low valence (LALV), low arousal/middle valence (LAMV), low arousal/high valence (LAHV), middle arousal/low valence (MALV), middle arousal/middle valence (MAMV), middle arousal/high valence (MAHV), high arousal/low valence (HALV), high arousal/middle valence (HAMV), and high arousal/high valence (HAHV).

#### Stimulation by Dance and Mime

**FIGURE 1:**
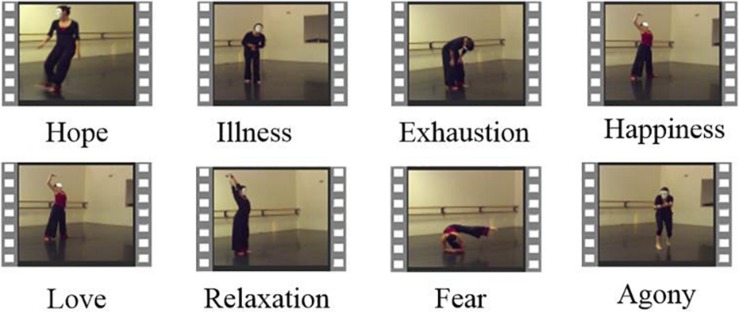
Videos on eight emotion themes.

We used the video resource mentioned in the literature (shown in Figure 1; Tipper et al., 2015). The videos expressed eight emotion themes, including four danced themes (happiness, hope, fear, and agony) and four pantomimed themes (love, relaxation, illness, and exhaustion). The emotion theme refers to the emotional content expressed by dancers in the video with their physical movements. Two dancers performed each emotion theme. Each dance or mime performance segment was recorded from two different shooting angles, so each emotion had four distinct videos. The video set included 32, 10 s videos. In the video, performers wore expressionless white masks. They conveyed emotional information though gestural whole-body movement, without facial expressions.

Twelve participants (seven females, five males) with little exposure to EEG experiments participated in the study. All participants were right-handed, and they had an average age of 22.3 years (variance, 3.3). During the experiment, the eight themes alternated. The participants indicated whether they ever had the desire to move.

**FIGURE 2:**
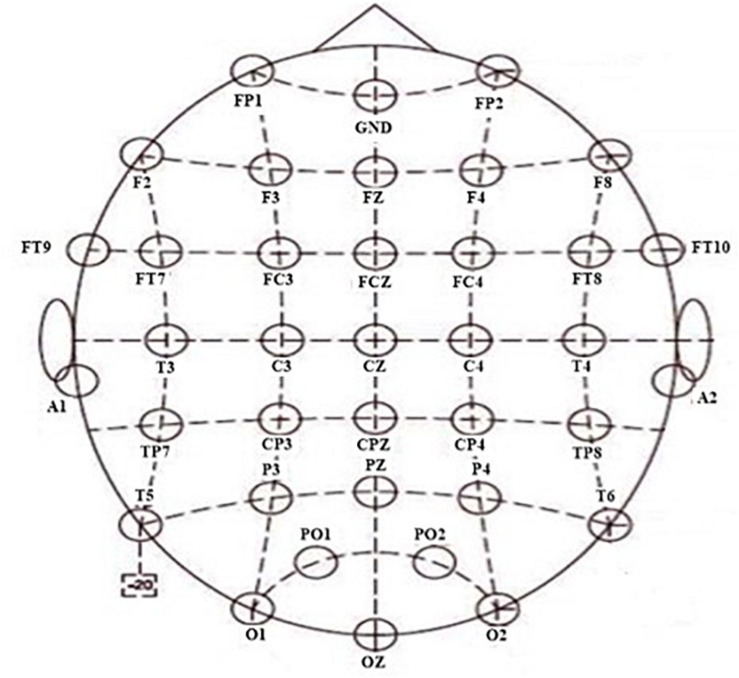
Layout of the 34 electrodes.

The NuAmps system (NeuroScan Inc. Sterling, United States) was used to record the EEG signals. The sampling rate was 1,000 Hz. For all electrodes, impedances were kept below 5 kΩ. From 40 channels, the EEG signals of 34 electrodes were used, with a ground electrode at Fz and reference electrode A1. The horizontal and vertical electrooculograms (EOGs) were recorded using four additional electrodes. The layout of the cap is according to the extended 10/20 system (shown in Figure 2). Software scan 4.5 was performed, with online EOG artifact rejection. A 50 Hz notch filter was used to suppress line noise. The cap of the NuAmps EEG amplifier has 34 EEG electrodes. The layout of the cap was based on the extended 10/20 system. The distribution of all electrodes in 10 brain regions was as follows: the electrodes FP1 and FP2 were distributed in the prefrontal lobe and the electrodes F7, F3, Fz, F4, and F8 distributed in the frontal lobe. The central region contained C3, Cz, and C4. The frontal lobe–central region contained FT9, FT7, FC3, FCz, FC4, FT8, and FT10. The electrodes in the temporal lobe were T3, T4, T5, and T6. The electrodes in the temporal–parietal lobe were TP7 and TP8. The parietal lobe contained P3, PZ, and P4. The electrodes CP3, CPz, and CP4 were distributed in the central region-parietal lobe. The parietal–occipital lobe contained PO1 and PO2. The occipital lobe contained O1, O2, and OZ.

### Connectivity Analysis: Bi-LSTM-GC Model

**FIGURE 3:**
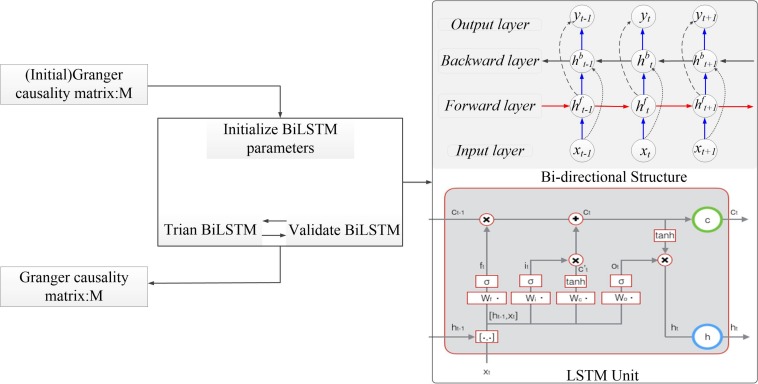
The algorithm structure of the bidirectional long short-term memory Granger causality (bi-LSTM-GC).

In this section, we introduce the bi-LSTM-GC model in detail (Figure 3). Multivariate GC can only capture linear relationships in a time series, whereas brain information transmission is nonlinear. The GC method, combined with neural networks, could be used to deal with the problem of nonlinearity and varying-length signal delays (Montalto et al., 2014; Wang et al., 2018a). An RNN is a kind of cyclic neural network that is based on an ordinary, multilayer backpropagation (BP) neural network (Zhao et al., 2019). It adds horizontal connections among the hidden layer units. Through a weight matrix, the value of a neuron in the previous time series can be transferred to the current neuron unit, so that the neural network has memory function. Basic LSTM units contain an input gate, an output gate, and a forget gate. These gates can be updated using historical information. At each time step *t*, hidden state *h*^*t*^ is updated by current data at the same time step *x*^*t*^, the hidden state at the previous time step *h*^*t*–1^, the input gate *i*^*t*^, the forget gate *f*^*t*^, the output gate *o*^*t*^, and a memory cell *c*^*t*^. For the input gate at time step *t*, *W*^i^ is the input weight, *V*^i^ is the hidden layer weight for previous time step *h*^*t*–1^, and *b*^i^ is the bias term. For the forget gate at time step *t*, *W*^f^ is the input weight, *V*^f^ is the hidden layer weight, and *b*^f^ is the bias term. For the output gate at time step *t*, *W*^o^ is the input weight, *V*^o^ is the input layer weight, and *b*^o^ is the bias term. For memory cell, *W*^c^ is the input weight, *V*^c^ is the input layer weight, and *b*^c^ is the bias term. σ is the sigmoid function. Tan*h* is the hyperbolic tangent function. The following updating equations are given as follows:

(1) $$\begin{array}{c} i^{t}=\sigma \left(W^{i}x^{t}+V^{i}h^{t-1}+b^{i}\right)\\ f^{t}=\sigma \left(W^{f}x^{t}+V^{f}h^{t-1}+b^{f}\right)\\ o^{t}=\sigma \left(W^{o}x^{t}+V^{o}h^{t-1}+b^{o}\right)\\ c^{t}=f^{t}⊙c^{t-1}+i^{t}⊙\tanh\left(W^{c}x^{t}+V^{c}h^{t-1}+b^{c}\right)\\ h^{t}=o^{t}⊙\tan h\left(c^{t}\right) \end{array}$$

Bi-LSTM networks (Schuster and Paliwal, 1997) were designed to capture sequential data and maintain contextual features from past to future. Unlike LSTM networks, bi-LSTM networks have two parallel layers propagating in two directions. The forward and backward pass of each layer is carried out similarly as in RNNs (Ma et al., 2019). Bidirectional LSTMs include forward and backward ways, with two separate hidden layers. They then feed-forward to the same output layer. The following equations define the hidden layer function (Zhao et al., 2017). The forward process equations were as follows:

(2) $$\begin{array}{c} {\vec{i}}^{t}=\sigma \left({\vec{W}}^{i}{\vec{x}}^{t}+{\vec{V}}^{i}{\vec{h}}^{t-1}+{\vec{b}}^{i}\right)\\ {\vec{f}}^{t}=\sigma \left({\vec{W}}^{f}{\vec{x}}^{t}+{\vec{V}}^{f}{\vec{h}}^{t-1}+{\vec{b}}^{f}\right)\\ {\vec{o}}^{t}=\sigma \left({\vec{W}}^{o}{\vec{x}}^{t}+{\vec{V}}^{o}{\vec{h}}^{t-1}+{\vec{b}}^{o}\right)\\ {\vec{c}}^{t}={\vec{f}}^{t}⊙{\vec{c}}^{t-1}+{\vec{i}}^{t}⊙\tan h\left({\vec{W}}^{c}{\vec{x}}^{t}+{\vec{V}}^{c}{\vec{h}}^{t-1}+{\vec{b}}^{c}\right)\\ {\vec{h}}^{t}={\vec{o}}^{t}⊙\tan h\left({\vec{c}}^{t}\right) \end{array}$$

The backward process equations were as follows:

(3) $$\begin{array}{c} {\overset{\leftarrow }{i}}^{t}=\sigma \left({\overset{\leftarrow }{W}}^{i}{\overset{\leftarrow }{x}}^{t}+{\overset{\leftarrow }{V}}^{i}{\overset{\leftarrow }{h}}^{t-1}+{\overset{\leftarrow }{b}}^{i}\right)\\ {\overset{\leftarrow }{f}}^{t}=\sigma \left({\overset{\leftarrow }{W}}^{f}{\overset{\leftarrow }{x}}^{t}+{\overset{\leftarrow }{V}}^{f}{\overset{\leftarrow }{h}}^{t-1}+{\overset{\leftarrow }{b}}^{f}\right)\\ {\overset{\leftarrow }{o}}^{t}=\sigma \left({\overset{\leftarrow }{W}}^{o}{\overset{\leftarrow }{x}}^{t}+{\overset{\leftarrow }{V}}^{o}{\overset{\leftarrow }{h}}^{t-1}+{\overset{\leftarrow }{b}}^{o}\right)\\ {\overset{\leftarrow }{c}}^{t}={\overset{\leftarrow }{f}}^{t}⊙{\overset{\leftarrow }{c}}^{t-1}+{\overset{\leftarrow }{i}}^{t}⊙\tan h\left({\overset{\leftarrow }{W}}^{c}{\overset{\leftarrow }{x}}^{t}+{\overset{\leftarrow }{V}}^{c}{\overset{\leftarrow }{h}}^{t-1}+{\overset{\leftarrow }{b}}^{c}\right)\\ {\overset{\leftarrow }{h}}^{t}={\overset{\leftarrow }{o}}^{t}⊙\tanh\left({\overset{\leftarrow }{c}}^{t}\right) \end{array}$$

In the present study, the bi-LSTM model and GC estimation were combined to propose a bidirectional LSTM-GC time series analysis algorithm. The model used sequential data with a variable-transmission time lag as inputs and used the LSTM model to learn information flow from data. Using these methods, we were able to estimate the complex dependency, such as nonlinearity and variable delay, in multivariate time series.

### Simulation Model

**FIGURE 4:**
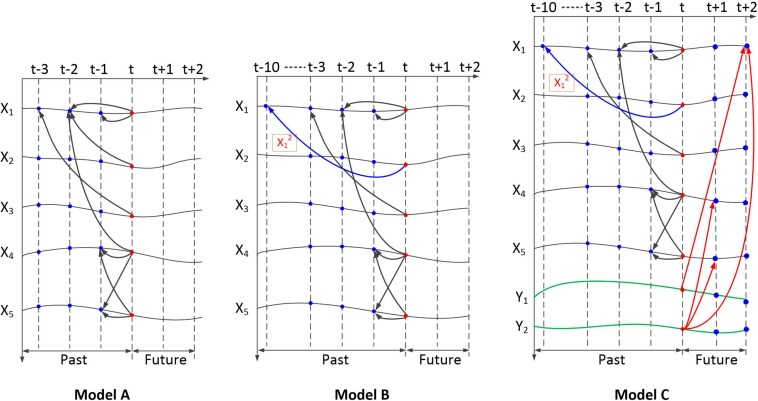
Dependency of the simulation signals generated by the three models. Model A is a multivariate linear model. Model B is a multivariate nonlinear model with varying lag lengths. Model C is a multivariate nonlinear model with varying lag lengths and bidirectional time dependency.

Three paradigms are included in the simulation test. These include linear simulations (model A), nonlinear simulations with varying lag lengths (model B), and bidirectional nonlinear simulations with varying lag lengths (model C). The dependency relationships within these models are shown in Figure 4.

According to the temporal and quantity relationship in model A (Baccalá and Sameshima, 2001), five signals are generated, as follows:

(3) $$\begin{array}{c} x_{1}\left(t\right)=0.95\sqrt{2}x_{1}\left(t-1\right)-0.9025x_{1}\left(t-2\right)+\varepsilon_{1}\left(t\right)\\ x_{2}\left(t\right)=0.5x_{1}\left(t-2\right)+\varepsilon_{2}\left(t\right)\\ x_{3}\left(t\right)=-0.4x_{1}\left(t-3\right)+\varepsilon_{3}\left(t\right)\\ x_{4}\left(t\right)=-0.5x_{1}\left(t-2\right)+0.25\sqrt{2}x_{4}\left(t-1\right)\\ +0.25\sqrt{2}x_{5}\left(t-1\right)+\varepsilon_{4}\left(t\right)\\ x_{5}\left(t\right)=-0.25\sqrt{2}x_{4}\left(t-1\right)+0.25\sqrt{2}x_{5}\left(t-1\right)+\varepsilon_{5}\left(t\right) \end{array}$$

Equation (3) presents the Gaussian noise with zero mean and unit variance. The length of the time series was set to 5,000. The ground truth of the simulation data contains five directional dependency pairs: 1→2, 1→3, 1→4, 4→5, and 5→4. The dependency relationship of the five signals is shown in Figure 4 (model A).

In the simulation of nonlinear connected signals with varying lag lengths, we adopt the nonlinear equations (model B) as follows:

(4) $$\begin{array}{c} x_{1}\left(t\right)=0.95\sqrt{2}x_{1}\left(t-1\right)-0.9025x_{1}\left(t-2\right)+\varepsilon_{1}\left(t\right)\\ x_{2}\left(t\right)=0.5x_{1}^{2}\left(t-10\right)+\varepsilon_{2}\left(t\right),\\ x_{3}\left(t\right)=-0.4x_{1}\left(t-3\right)+\varepsilon_{3}\left(t\right)\\ x_{4}\left(t\right)=-0.5x_{1}\left(t-2\right)+0.25\sqrt{2}x_{4}\left(t-1\right)\\ +0.25\sqrt{2}x_{5}\left(t-1\right)+\varepsilon_{4}\left(t\right)\\ x_{5}\left(t\right)=-0.25\sqrt{2}x_{4}\left(t-1\right)+0.25\sqrt{2}x_{5}\left(t-1\right)+\varepsilon_{5}\left(t\right) \end{array}$$

Most of the settings are the same as Equation (3). The modified part occurs in dependency 1→2. The second-order functions and settings of varying length lags involving long delays are used.

Sequence *x*_2_ (*t*) depends on *x*_1_ (*t*), with a 10-point time delay.

For the third test, we partially modify the signal generation model. Bidirectional dependency is set as linear dependency with short delays. In the simulation of nonlinear dependency signals with varying lag lengths, we adopt nonlinear equations (model C) as follows:

(5) $$\begin{array}{c} x_{1}\left(t\right)=0.95\sqrt{2}x_{1}\left(t-1\right)-0.9025x_{1}\left(t-2\right)+\varepsilon_{1}\left(t\right)\\ x_{2}\left(t\right)=0.5x_{1}^{2}\left(t-10\right)+\varepsilon_{2}\left(t\right)\\ x_{3}\left(t\right)=-0.4x_{1}\left(t-3\right)+\varepsilon_{3}\left(t\right)\\ x_{4}\left(t\right)=-0.5x_{1}\left(t-2\right)+0.25\sqrt{2}x_{4}\left(t-1\right)\\ +0.25\sqrt{2}x_{5}\left(t-1\right)+\varepsilon_{4}\left(t\right)\\ x_{5}\left(t\right)=-0.25\sqrt{2}x_{4}\left(t-1\right)+0.25\sqrt{2}x_{5}\left(t-1\right)+\varepsilon_{5}\left(t\right)\\ y_{1}\left(\text{t}\right)=0.6x_{1}\left(t+2\right)+\varepsilon_{2}\left(t\right)\\ y_{2}\left(t\right)=-0.5x_{1}\left(t+2\right)+0.25\sqrt{2}x_{4}\left(t+1\right)\\ +0.25\sqrt{2}x_{5}\left(t+1\right)+\varepsilon_{4}\left(t\right) \end{array}$$

Based on model B, *y*_1_ and *y*_2_ were added, whereby *y*_1_(*t*) forward depended on *x*_1_(*t* + 2) and *y*_2_(*t*) forward depends on *x*_1_(*t* + 2), *x*_4_(*t* + 1), and *x*_5_(*t* + 1). The dependency relationship of the seven signals is shown in Figure 4. In this simulation, the length of the time series was set to 5,020. Because model C could not generate the first points of *y*_1_ and *y*_2_, they are set as random numbers.

## Results

To demonstrate the advantages of the bi-LSTM-GC method, we herein present the comparison results of a dependency detection among bi-LSTM-GC, neural network GC (Montalto et al., 2014), and RNN-GC (Wang et al., 2018b). We implemented NN-GC using an open-source MATLAB toolbox (Montalto et al., 2014). For RNN-GC, the Open Source code^1^ is adopted for the test. The simulation dataset included time series with linear dependency, as well as nonlinear dependency that contained varying lag lengths and long delays. Next, we analyzed the brain connectivity of emotion–movement regulation with the proposed method.

### Simulations

The neural network GC (Montalto et al., 2014) presents a Granger causality measure that uses feedforward neural networks for prediction. One hidden layer is used and the number of hidden units is set to two thirds of the neuron number in the input layer. The parameter of model order is set to 5 for model A/B and 7 for model C. The lengths of the time series were set to 5,000 (model A/B) and 5,020 (model C). The NN-GC also reshapes the multivariate time series matrix (5 × 5,000 and 7 × 5,020) into a vector. This procedure loses the temporal structure of data, thus leading to inaccurate predictions. For RNN-GC, the LSTM model with one hidden layer is adopted and there are 10 units in the hidden layer. The maximum sequence length of LSTM is set to 20. In order to preserve the temporal structure of the data, the data in the time steps are recurrently fed to the network. This mode of input is also used in the test of bi-LSTM-GC.

**FIGURE 5:**
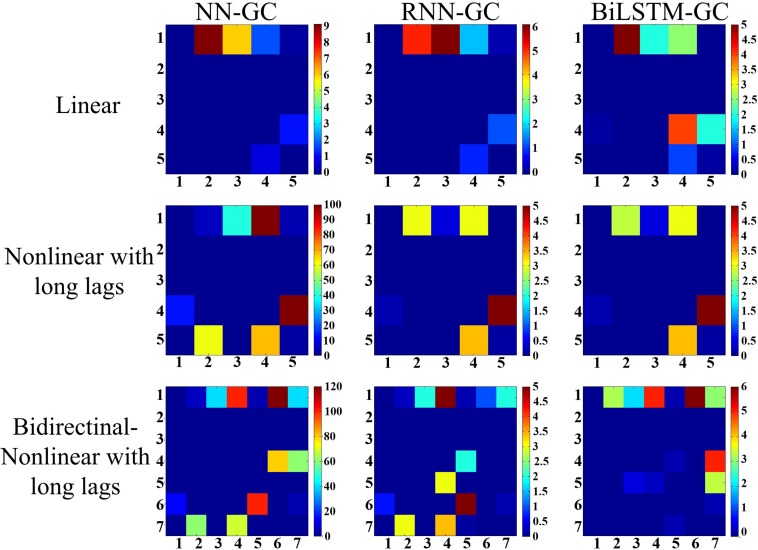
Results derived from three simulation models are illustrated. (1) Model A: linear simulations, (2) model B: nonlinear simulations with varying lag lengths, (3) model C: nonlinear simulations with bidirectional dependency and varying lag lengths. The *colored matrices* represent the distribution of the overall directional dependencies. The term “*Mij*” refers to the directional dependency from sequence *i* to sequence *j*. *Different colors* represent dependence intensity, and *lighter colors* indicate stronger dependencies. All the results are averaged over 10 trials, and self-dependencies are not included.

In the test result from model A (Figure 5, first row), RNN-GC gave the most accurate results. All the dependencies presupposed in model A were correctly captured, with no false alarms. Dependencies were successfully detected in some cases using bi-LSTM-GC, but invalid detections also occurred. In the test result from model B (Figure 5, second row), RNN-GC and bi-LSTM-GC captured all dependencies without any false detections. Using NN-GC, all dependencies were detected, but the detections were obscured by noise from false detections. Moreover, invalid detections occurred in the tests of signals with long time delays. Using model C, two forward dependencies were added: (*y*_1_(*t*) → *x*_1_(*t* + 2) and *y*_2_(*t*) → *x*_1_(*t* + 2), *x*_4_(*t* + 1), *x*_5_(*t* + 1). In the test result from model C (Figure 5, third row), bi-LSTM-GC exhibited the best accuracy, with all the dependencies being correctly captured and without false detections. RNN-GC achieved the detection of nonlinear and long delays well, but it could not deal with the detections of forward dependency. Here, the forward dependency means that the value of a variable at a certain time is related to the subsequent values. The detections were obscured by noise from false detections.

### Brain Connectivity in Emotion–Motion Regulation

The direction and intensity of information flow were quantitatively estimated to explore the causality of activation among different brain regions in the state of emotion–movement regulation.

#### Stimulation by Music (DEAP Dataset)

Each participant contributed 40 segments of EEG data, each of which had a V–A tag. We separated EEG data segments with the same V–A tag into one group. Before connectivity analysis, we used 10-order Chebyshev I bandpass filters to extract five characteristic frequencies, namely, delta (0.5–3 Hz), theta (4–7 Hz), alpha (8–13 Hz), beta (13–30 Hz), and gamma (31–50 Hz). The data preprocessing included covariance stationarity, detrending, and demeaning.

The data from seven of the participants did not meet the test criteria, so the data of 25 participants were ultimately used in the bi-LSTM-GC calculation. After bi-LSTM-GC analysis, we separately confirmed the characteristic frequencies of all effective directional dependencies of each participant in different emotion states. Next, all the bi-LSTM-GC matrices with the same V–A tag and in the same characteristic frequency were averaged. Thus, the bi-LSTM-GC matrices related to specific emotions were obtained. The stable dependencies between EEG signals were extracted using the *t* test (*p* < 0.05; Table 1). In the table, “null” means that the bi-LSTM-GC estimator did not produce verifiable dependency from any participants or at any frequency.

**TABLE 1:** DEAP: stable dependencies (more than 10 occurrences) in all participants’ data.

Emotion	Arousal–Valence	Dependency	Frequency (Hz)
Happy	HAHV	Fp1→T7	4–13
		F7→Cz	4–13
		AF4→FC6	0.5–8
		C4→F3	4–7
Pleased	MAHV	Null	
Relaxed	LAHV	Null	
Excited	HAMV	F5→CP5	4–30
		AF3→C3	13–30
Neutral	MAMV	Null	
Calm	LAMV	Null	
Distressed	HALV	F6→Cz	4–30
		F2→T8	4–13
		Fp2→CP2	0.5–8
		F1→CP1	4–13
Miserable	MALV	Null	
Depressed	LALV	Null	

#### Stimulation by Dance and Mime

In the present experiment, 12 participants were recruited. Preprocessing was carried out as described in the literature (Li et al., 2018). For preprocessing, software scan 4.5 performed online EOG artifact rejection. A 50 Hz notch filter was used to suppress line noise. For raw EEG signals, the left preprocessing steps were manually performed in software scan 4.5, which include linear derivation, DC offset correction, merge task date, blink reject, movement artifact reject, and baseline correct. We analyzed the EEG signals collected during video watching. For each trial EEG, there were two timestamps to identify the beginning and end of the video. After pre-analysis, the data from nine participants met the test criteria. The directional dependency detected in one participant’s EEG signal has four types of information: first, the emotion theme of the video the participant watched during the experiment; second, the way the dancer expressed the emotion theme in the video, such as dancing; and the electrodes and frequency involved in the directional dependency.

The stable dependencies between electrodes were extracted using the *t* test (*p* < 0.05; Table 2). Through the estimation with the bi-LSTM-GC algorithm, the entire directional dependencies were detected from all participants’ EEG signals. Table 2 shows the stable dependencies, accumulated more than 10 occurrences over all participants: first, the emotion theme of the video the participant watched during the experiment; second, the way the dancer expressed the emotion theme in the video, such as dancing; and the electrodes and frequency involved in the directional dependency. Among the eight emotion themes, no stable dependency was detected in the stimulation of “Happy/Dance,” “Hopeful/Dance,” “Love/Mime,” “Relaxed/Mime,” and “Exhausted/Mime.” In the stimulation of “Fearful/Dance,” “Agony/Dance,” and “Ill/Mime,” the stable dependencies evidently increased. These stable dependencies centrally appeared in the frontal and central motor area in low frequencies (0.5–30 Hz).

**TABLE 2:** Experimental dataset: stable dependencies (more than 10 occurrences) in all participants’ data.

Emotion	Form	Dependency	Frequency (Hz)
Happy	Dance	Null	
Hopeful	Dance	Null	
Fearful	Dance	Fp1→C3	4–13
		FT7→Cz	0.5–8
		Cz→F8	4–30
		F3→C4	13–30
		Fz→C4	4–13
Agony	Dance	F4→C4	8–30
		CP3→F4	8–13
		C3→F8	4–13
		Fp2→Cz	4–13
		Fp2→CP4	13–30
		Fp1→CP4	4–7
Love	Mime	Null	
Relaxed	Mime	Null	
Ill	Mime	C3→FC3	13–30
Exhausted	Mime	Null	

**FIGURE 6:**
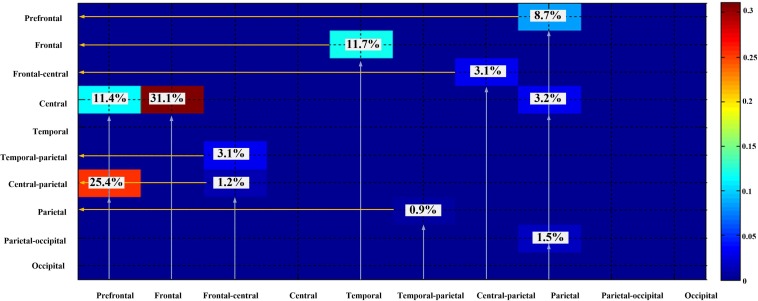
Dependencies among different brain regions.

According to the results of the entire directional dependencies (Figure 6), 34.1% of all stable directional dependencies were from the frontal lobe to the central region, 25.4% were from the prefrontal lobe to the central region–parietal lobe, 11.7% were from the temporal lobe to the frontal lobe, 10.2% were from the prefrontal lobe to the central region, and 8.7% were from the parietal lobe to the prefrontal lobe.

## Discussion

At the beginning of this paper, we hypothesize that the emotion cortex interacts with the motor cortex during the mutual regulation of emotion and movement. To test this hypothesis, a brain connectivity analysis method is proposed, named bidirectional long short-term memory Granger Causality (bi-LSTM-GC). Therefore, this section contains two parts. The first part is about brain connectivity in emotion–movement regulation. The other part is the verification of the effectiveness and stability of bi-LSTM-GC.

### Motion-Affective Connectivity

This study is an interdisciplinary study combining emotional cognition, motor cognition, and brain connectivity. To explore the neural information characteristics of the mutual regulation of emotion and movement, a novel method was proposed in this paper. Brain connectivity analysis is a widely used method for studying the dynamic or static characteristics of brain neural information. Coherence was used as a connectivity index to estimate the functional brain connectivity on EEG signals for all the multiple emotions (Chandra et al., 2016). The results indicated that gender differences cause special differences in brain connectivity (Yin et al., 2018). Based on connectivity analysis, a general emotion pathway was detected. Considered as a common pathway within the general emotion network, this general emotion pathway was involved in shared basic psychological processes across emotions (Huang et al., 2018). For functional MRI (fMRI) study, the emotions have discrete neural bases characterized by specific, distributed activation patterns in widespread cortical and subcortical circuits (Saarimäki et al., 2018). Locally differentiated engagement of these globally shared circuits defines the unique neural fingerprint activity pattern and the corresponding subjective feeling associated with each emotion (Saarimäki et al., 2018). The impaired facial emotion perception has been found in individuals with negative schizotypy (NS), and the corresponding change in brain functional connectivity was explained in detail (Wang et al., 2018a).

All the above research only focused on the emotion cognition. Our interest was the brain connectivity during the mutual regulation of emotion and movement. According to the literatures (Selma Aybek et al., 2015; Tipper et al., 2015; Hua et al., 2018; Neill et al., 2019), synchronous activation of the frontal lobe and motor cortex occurs during emotion recognition. In the present research, quantitative calculation found similar results in the state of emotion–movement regulation. To the best of our knowledge, this method was proposed for the first time. Using the DEAP dataset, nine tags were defined to identify emotion states, according to a valence–arousal model. The “happy” tag (high arousal/high valence) mainly occurred in four groups with stable dependencies, which existed across the prefrontal lobe, frontal lobe, and central region (0.5–13 Hz). The distress tag (high arousal/low valence) obtained the largest numbers of stable dependencies, which existed across the prefrontal lobe, frontal lobe, and central region–parietal lobe (0.5–30 Hz). For the experiment dataset, two forms were used to express emotional themes: dance and mime. Here, the “arousal–valence” tags were not used. Only the basic identification was used to label all emotions. The most important stable dependencies were detected for the emotions of agony and fear. The directionality of stable dependencies in the EEG signals was mainly from the frontal lobe to the central region, from the prefrontal lobe to the central region–parietal lobe, from the temporal lobe to the frontal lobe, from the prefrontal lobe to the central region, and from the parietal lobe to the prefrontal lobe.

### Bi-LSTM-GC

The idea of causality was first put forward by Norbert Wiener (Jafari-Mamaghani, 2014). The econometrician Clive Granger (1969) then made it computable in terms of linear vector autoregressive models. GC is increasingly being applied to multi-electrode neurophysiological and functional imaging data to characterize directional interactions between neurons and brain regions (Wen et al., 2013). In addition, most estimation algorithms that use regression estimation require a fixed order, so they only process signals with fixed time lag lengths. Montalto et al. (2014) proposed NN-GC to improve the ability of GC in the aspect of processing variable time lag lengths. Here, a feed-forward neural network is introduced to fit the regression parameters in the GC model; this makes the model effective with nonlinear dependencies in sequential data. However, most existing nonlinear methods can barely detect dependencies with variable time delays. As a special kind of RNN, LSTM neural networks (Hochreiter and Schmidhuber, 1997) are efficient in modeling sequential data with bidirectional dependencies (Sundermeyer et al., 2015).

To make GC estimators effectively solve the problem of variable-length time lags and long transmission delays in sequential data, the LSTM-GC was proposed based on the LSTM and GC theory (Wang et al., 2018b). However, EEG signal transmission presents complex temporal and spatial characteristics, and the dependencies in the EEG signals present the bidirectional historical retrospective relationship. In terms of the time dimension, estimation algorithms must be able to handle bidirectional dependencies. In the present study, the bi-LSTM model and GC were combined to form a causality analysis method. The bi-LSTM is actually a three-layer stacked LSTM model used for the sequential data regression problem. The core idea behind the deep neural network is that inputs to the model should go through multiple nonlinear layers. When it comes to deep LSTMs, the input to the model can be passed through multiple LSTM layers. The hidden output of one LSTM layer is not only propagated through time but also used as the input data to the next LSTM layer. In the framework of bidirectional LSTM, every hidden layer receives an input sequence that consists of the output sequences of the forward and backward layers at the level below. In the test of the multivariate linear model, with or without variable time delay, bi-LSTM-GC showed comparable results. Furthermore, in the test of multivariate nonlinear signals containing bidirectional time dependencies, the proposed method showed better robustness.

In summary, the contribution of this paper is the proposal of a method for GC estimation based on bi-LSTM neural networks. For brain connectivity research, the paradigm of emotion–movement regulation can also be extended to other multi-cognitive conditions. Hyperscanning is a kind of technique that simultaneously records neural activities from multiple interacting participants. The Granger causality (GC) is a very popular method for calculating the directional hyperlink in hyperscanning study. The method of bi-LSTM-GC can be a new algorithm for hyperscanning.

## Data Availability Statement

The raw data supporting the conclusions of this manuscript will be made available by the authors, without undue reservation, to any qualified researcher.

## Ethics Statement

The studies involving human participants were reviewed and approved by the ethics committee at Xi’an Jiaotong University, China. The patients/participants provided their written informed consent to participate in this study.

## Author Contributions

TL was mainly responsible for designing experiments, data analysis, and writing the manuscript. GL was responsible for guiding the revision of the manuscript and put forward the suggestions for the algorithm design. TX was mainly responsible for organizing the experimental environment and equipment. JZ was mainly responsible for general guidance and project management.

## Conflict of Interest

The authors declare that the research was conducted in the absence of any commercial or financial relationships that could be construed as a potential conflict of interest.

## Footnotes

1 The Open Source code of the RNN-GC is available on GitHub at https://github.com/shaozhefeng/RNN-GC.
